# Supplementary material for: Exogenous Copper Application for the Elemental Defense of Rice Plants against Rice Leaffolder (Cnaphalocrocis medinalis)
Source: Plants (Basel). 2022 Apr 19;11(9):1104. doi: 10.3390/plants11091104 (PMC9099555; doi:10.3390/plants11091104)
Supplement: Supplementary file 1 [file plants-11-01104-s001.zip › plants-1683409-supplementary.pdf]

Supplementary Information

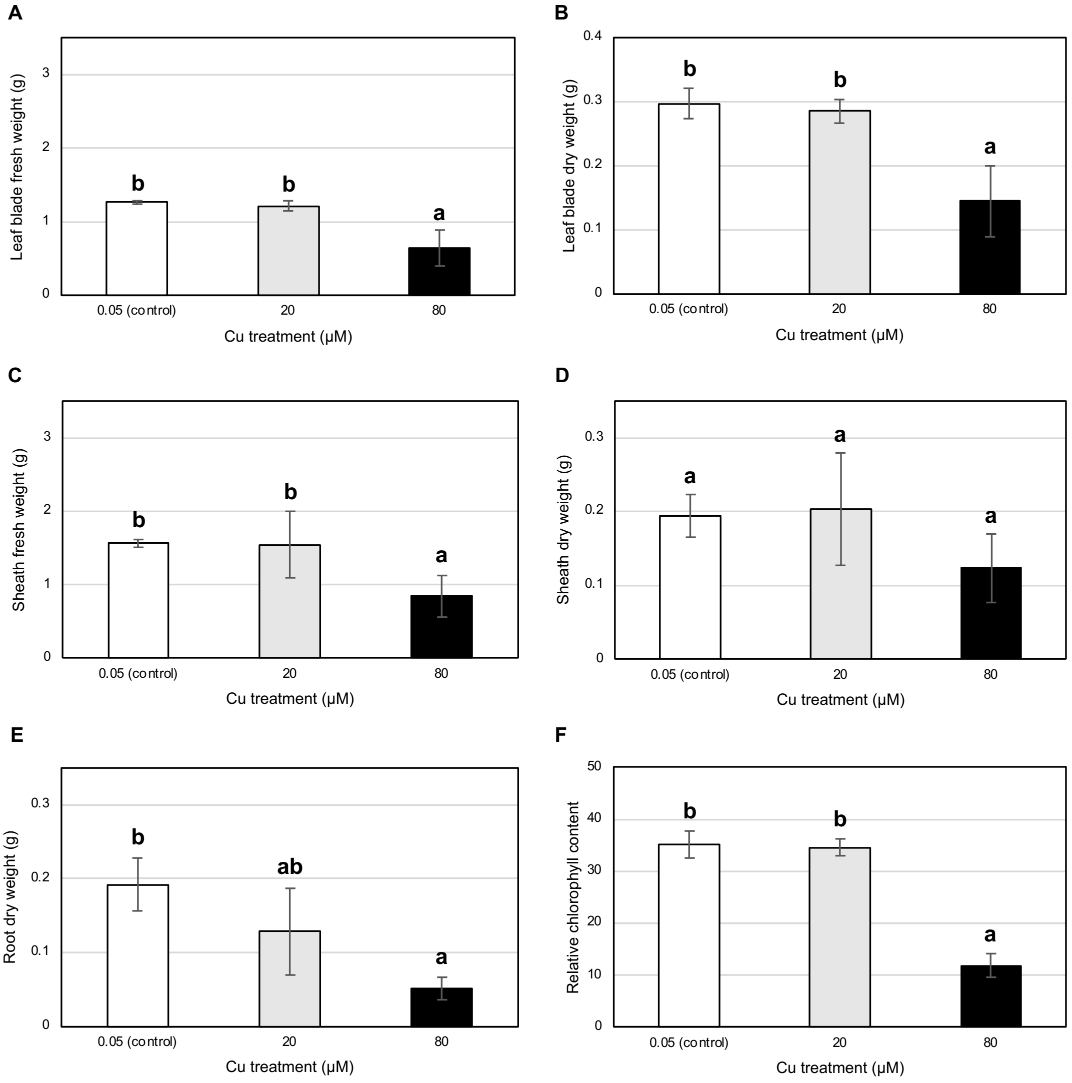

Figure S1. Effects of 20 and 80 μM Cu on the biomass and relative chlorophyll content of vegetative rice plants at 15 DAT. (**A**) Leaf blade fresh weight (FW), (**B**) leaf blade dry weight (DW), (**C**) sheath FW, (**D**) sheath DW, (**E**) root DW, and (**F**) relative chlorophyll content (SPAD) are shown. Data are means ± SD (tissue FW and DW, n = 4; relative chlorophyll content, n = 10). One-way ANOVA and Tukey's *post hoc* test were used, with different letters denoting a significant difference ($p < 0.05$).

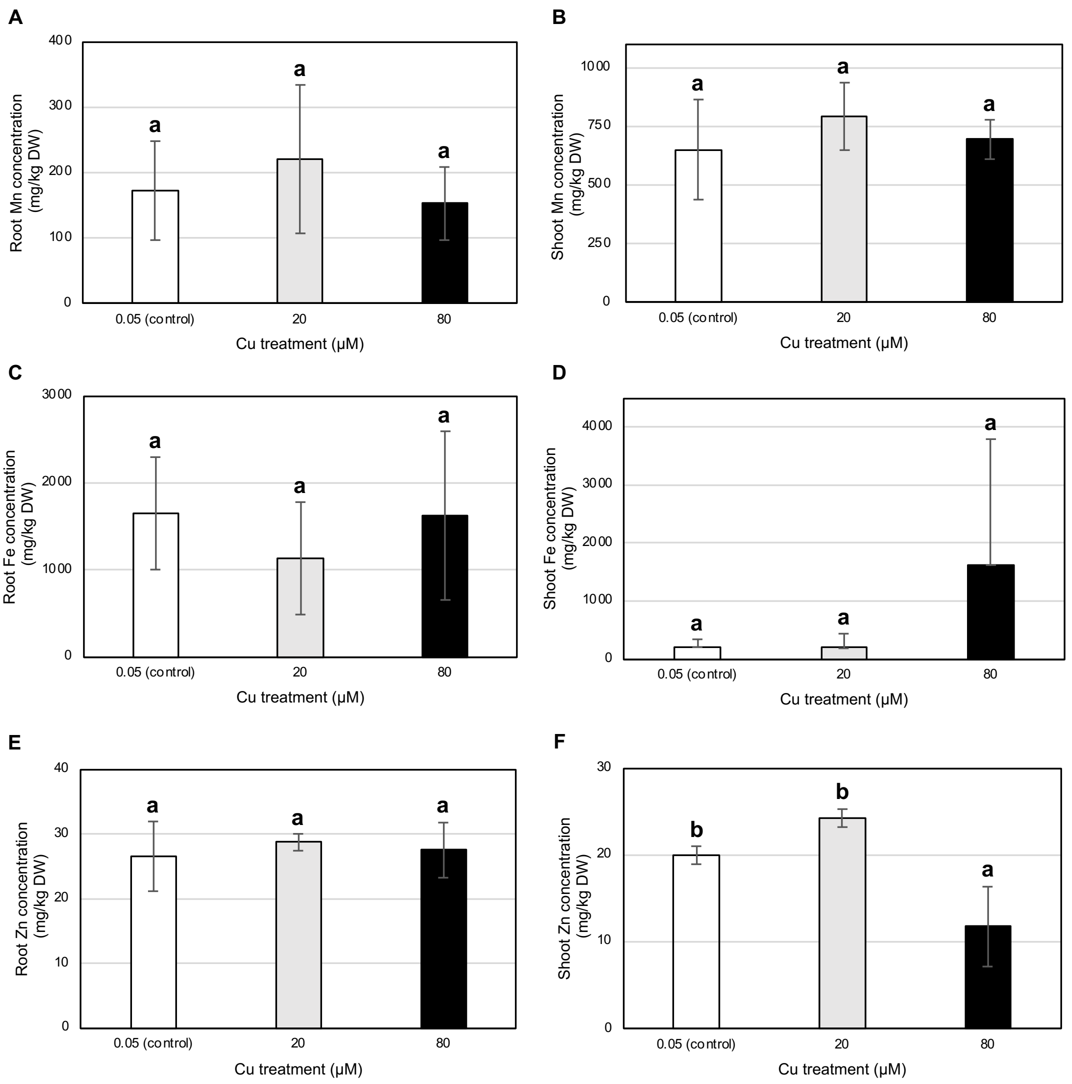

Figure S2. Effects of 20 and 80 μM Cu on the Mn, Fe and Zn micronutrient concentrations of vegetative rice plants at 15 DAT. (**A**) Root Mn, (**B**) shoot Mn, (**C**) root Fe, (**D**) shoot Fe, (**E**) root Zn, and (**F**) shoot Zn concentrations (mg/Kg DW) are shown as means ± SD (n = 4). One-way ANOVA and Tukey's *post hoc* test were used, with different letters denoting a significant difference ($p < 0.05$).

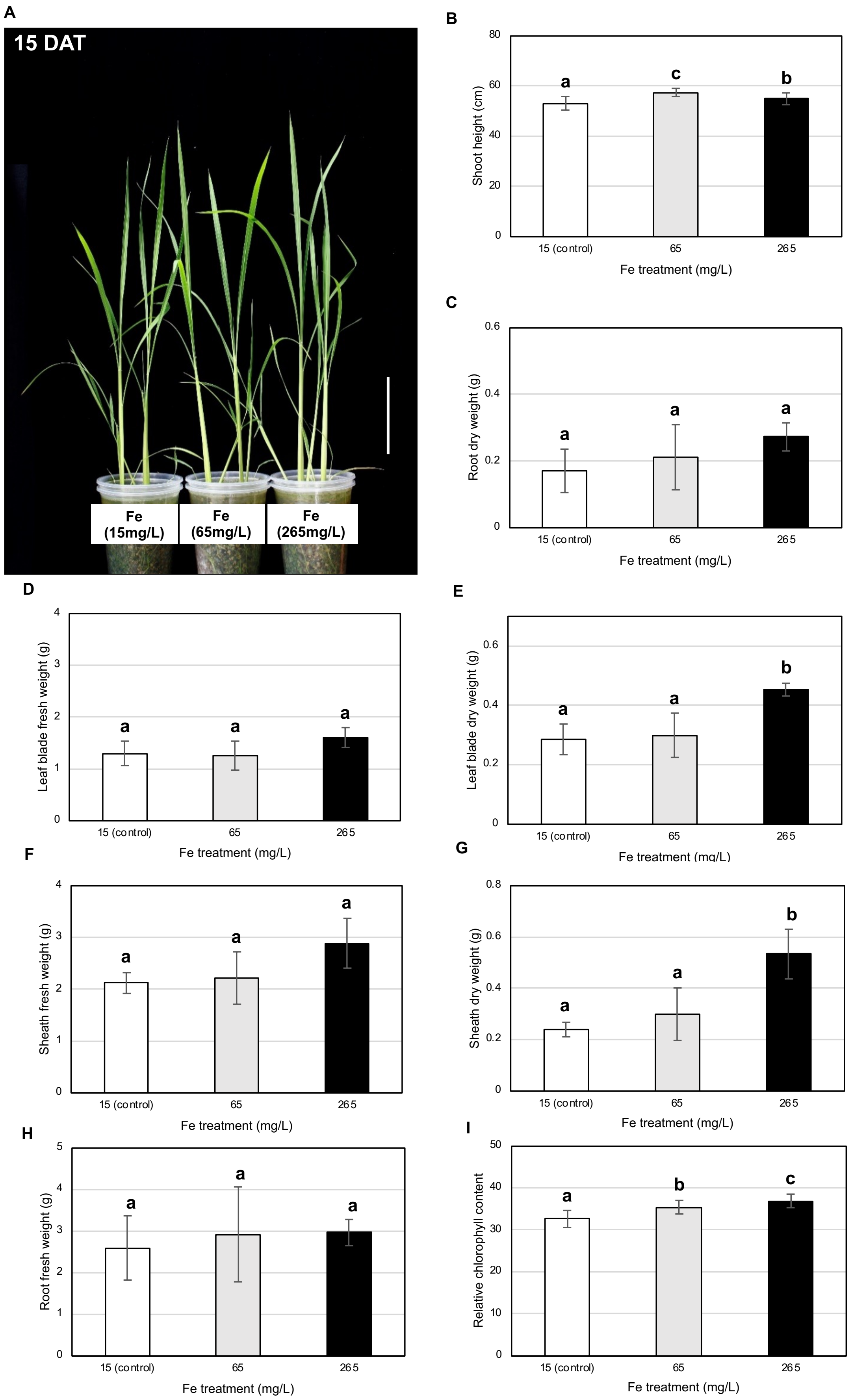
A
15 DAT
Fe (15mg/L)
Fe (65mg/L)
Fe (265mg/L)
B
Shoot height (cm)
Fe treatment (mg/L)
15 (control)
65
265
C
Root dry weight (g)
Fe treatment (mg/L)
D
Leaf blade fresh weight (g)
Fe treatment (mg/L)
E
Leaf blade dry weight (g)
Fe treatment (mg/L)
F
Sheath fresh weight (g)
Fe treatment (mg/L)
G
Sheath dry weight (g)
Fe treatment (mg/L)
H
Root fresh weight (g)
Fe treatment (mg/L)
I
Relative chlorophyll content
Fe treatment (mg/L)

Figure S3. Effects of 65 and 265 mg/L Fe on the physiology of vegetative rice plans at 15 DAT. (**A**) Shoot morphology (scale bar = 10 cm), (**B**) shoot height, (**C**) root dry weight (DW), (**D**) leaf blade fresh weight (FW), (**E**) leaf blade DW, (**F**) sheath FW, (**G**) sheath DW, (**H**) root FW, and (**I**) relative chlorophyll content (SPAD) are shown. Data are means ± SD (shoot height, n = 10; tissue FW and DW, n = 4; relative chlorophyll content, n = 10). One-way ANOVA and Tukey's *post hoc* test were used, with different letters denoting a significant difference ($p < 0.05$).

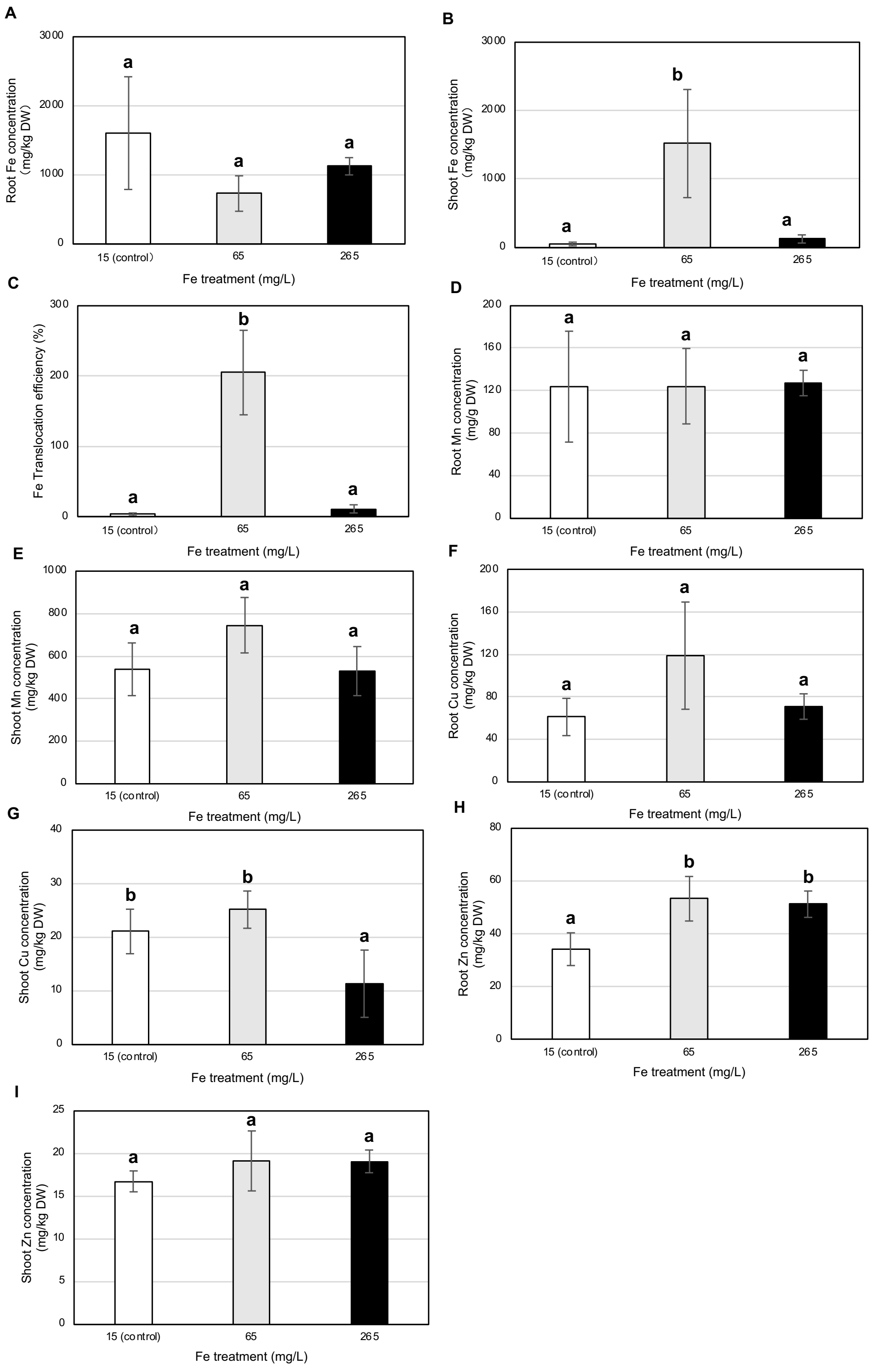

Figure S4. Effects of 65 and 265 mg/L Fe on the Fe, Mn, Cu and Zn micronutrient concentrations of vegetative rice plants at 15 DAT. (**A**) Root Fe, (**B**) shoot Fe, (**C**) Fe translocation efficiency from root to shoot, (**D**) root Mn, (**E**) shoot Mn, (**F**) root Cu, (**G**) shoot Cu, (**H**) root Zn, and (**I**) shoot Zn are shown as means ± SD (n = 4). One-way ANOVA and Tukey's *post hoc* test were used, with different letters denoting a significant difference ($p < 0.05$).

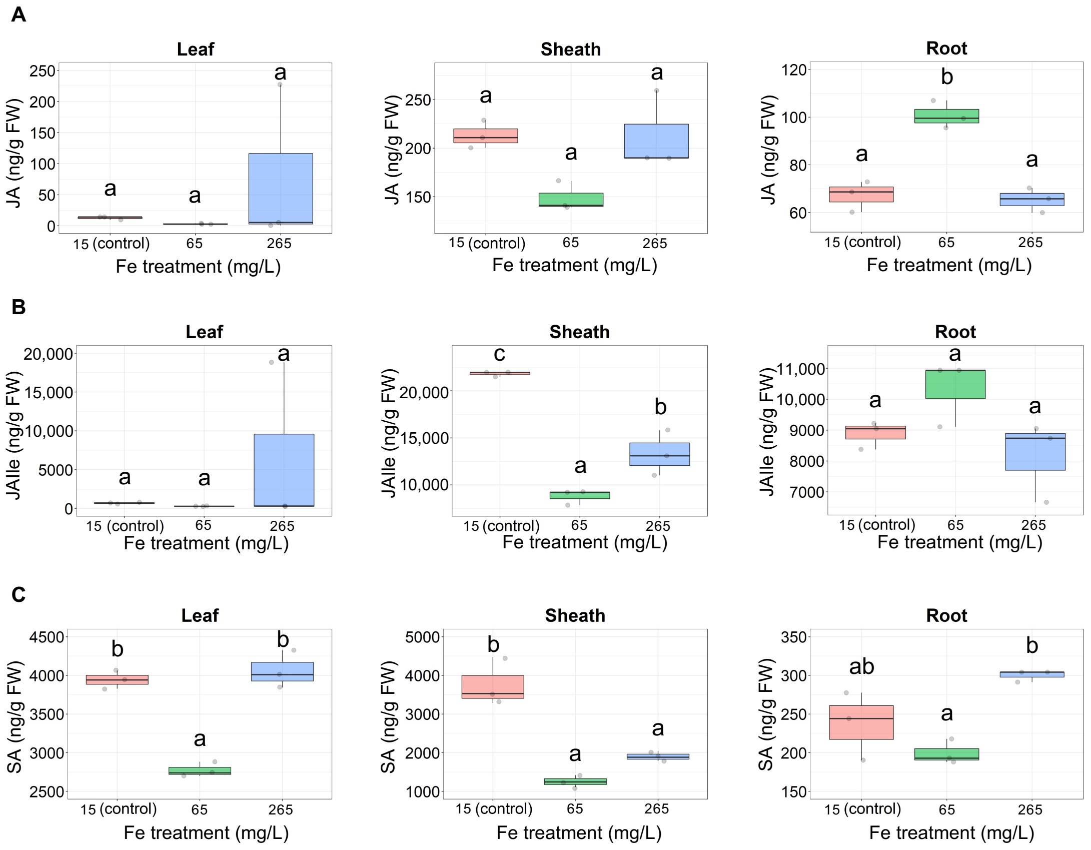

Figure S5. Effects of 65 and 265 mg/L Fe on defense-related phytohormones in rice tissues at 15 DAT. (**A**) Jasmonic acid (JA), (**B**) JA-isoleucine (JAIle) and (**C**) salicylic acid (SA) concentrations are shown in three tissues. Data are means ± SD (n = 3). One-way ANOVA and Tukey's *post hoc* test were used for phytohormone measurements in each tissue, with different letters denoting a significant difference ($p < 0.05$).

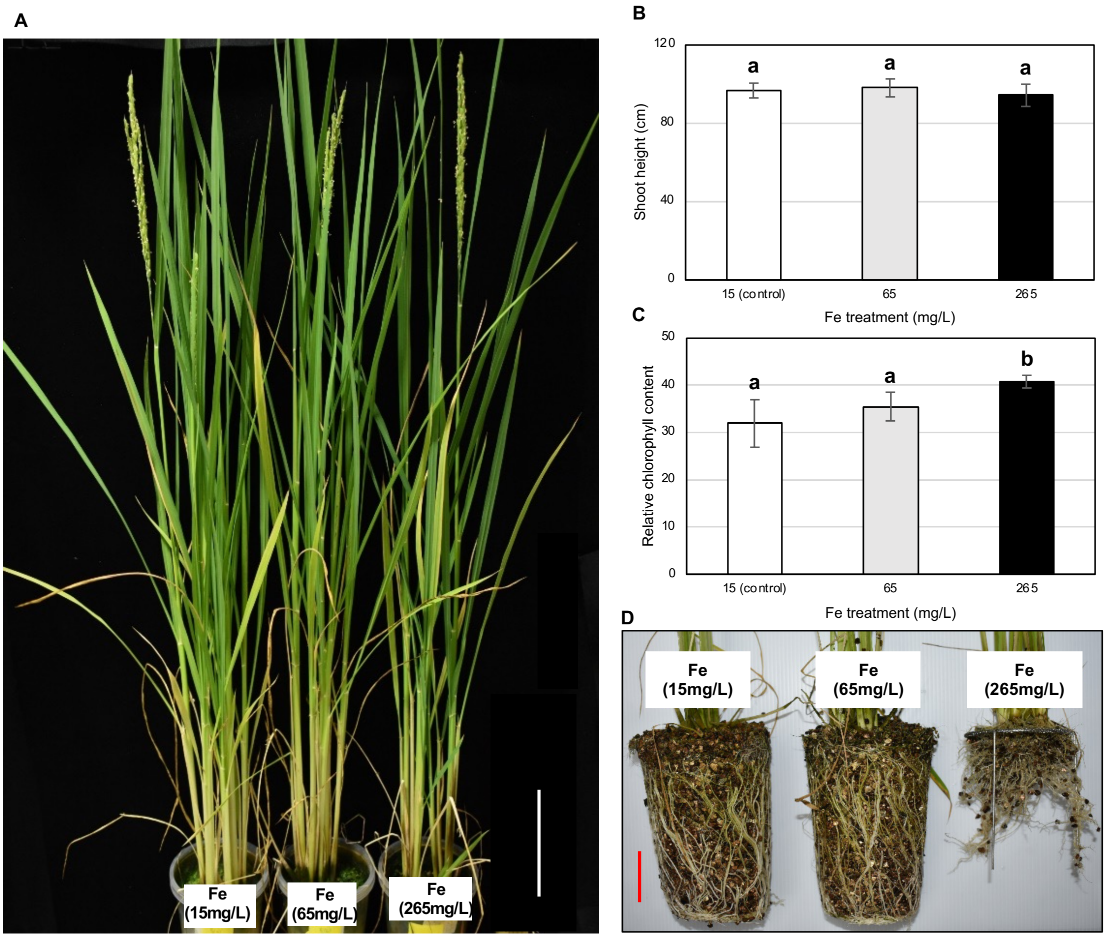

Figure S6. Effects of 65 and 265 mg/L Fe on the physiology of rice plants at the reproductive stage. (**A**) Shoot morphology during heading (scale bar = 10 cm), (**B**) shoot height, (**C**) relative chlorophyll content, and (**D**) root morphology (scale bar = 5 cm) are shown. Data are means ± SD (shoot height, n = 8; relative chlorophyll content, n = 4). One-way ANOVA and Tukey's *post hoc* test were used, with different letters denoting a significant difference ($p < 0.05$).
